# Immune modulation and increased neurotrophic factor production in multiple sclerosis patients treated with testosterone

**DOI:** 10.1186/1742-2094-5-32

**Published:** 2008-07-31

**Authors:** Stefan M Gold, Sara Chalifoux, Barbara S Giesser, Rhonda R Voskuhl

**Affiliations:** 1 Department of Neurology, Neuroscience Research Building 1, 635 Charles E. Young Drive South, University of California Los Angeles, CA, 90095, USA; 2 Cousins Center, 300 Medical Plaza, University of California Los Angeles, CA, 90095, USA

## Abstract

**Background:**

Multiple sclerosis is a chronic inflammatory disease of the central nervous system with a pronounced neurodegenerative component. It has been suggested that novel treatment options are needed that target both aspects of the disease. Evidence from basic and clinical studies suggests that testosterone has an immunomodulatory as well as a potential neuroprotective effect that could be beneficial in MS.

**Methods:**

Ten male MS patients were treated with 10 g of gel containing 100 mg of testosterone in a cross-over design (6 month observation period followed by 12 months of treatment). Blood samples were obtained at three-month intervals during the observation and the treatment period. Isolated blood peripheral mononuclear cells (PBMCs) were used to examine lymphocyte subpopulation composition by flow cytometry and *ex vivo *protein production of cytokines (IL-2, IFNγ, TNFα, IL-17, IL-10, IL-12p40, TGFβ1) and growth factors (brain-derived neurotrophic factor BDNF, platelet-derived growth factor PDGF-BB, nerve growth factor NGF, and ciliary neurotrophic factor CNTF). Delayed type hypersensitivity (DTH) skin recall tests were obtained before and during treatment as an *in vivo *functional immune measure.

**Results:**

Testosterone treatment significantly reduced DTH recall responses and induced a shift in peripheral lymphocyte composition by decreasing CD4+ T cell percentage and increasing NK cells. In addition, PBMC production of IL-2 was significantly decreased while TGFβ1 production was increased. Furthermore, PBMCs obtained during the treatment period produced significantly more BDNF and PDGF-BB.

**Conclusion:**

These results are consistent with an immunomodulatory effect of testosterone treatment in MS. In addition, increased production of BDNF and PDGF-BB suggests a potential neuroprotective effect.

**Trial Registration:**

NCT00405353

## Background

Multiple sclerosis (MS) has been considered a putative T cell mediated autoimmune disease. However, it is becoming increasingly clear that its pathology is far more complicated and is characterized by both an inflammatory component as well as a neurodegenerative process [1]. To date, the relationship between inflammation and the neurodegeneration in MS is unclear. Nevertheless, it is currently thought that potential new treatments should ideally have anti-inflammatory as well as neuroprotective properties [2].

Numerous models of autoimmune diseases including experimental autoimmune encephalomyelitis (EAE), diabetes in nonobese mice, thyroiditis, and adjuvant arthritis have been shown to be worsened by castration of male animals [3-8]. Furthermore, testosterone treatment ameliorates EAE [9]. These protective effects are thought to be mediated by testosterone's immunomodulatory properties such as decreasing the production of pro-inflammatory cytokines TNFα and IL-1β by macrophages [10] and monocytes [11] as well as increasing production of the anti-inflammatory cytokine IL-10 by T cells [12].

In addition, evidence supporting a neuroprotective effect of testosterone has been found in a variety of neurological diseases as well as in the cognitive decline associated with normal aging [13]. Testosterone is converted to estrogen in the brain by aromatase, and the neuroprotective properties of testosterone treatment *in vivo *may be due at least in part to this conversion. However, several *in vitro *studies have shown that testosterone can also be more directly neuroprotective [14]. Testosterone has been shown to protect spinal cord neurons in culture from glutamate-mediated toxicity, as well as to induce neuronal differentiation and increase neurite outgrowth [15]. Testosterone treatment also protected cultured neurons against beta-amyloid toxicity induced cell death [16].

While CNS infiltrating immune cells were once thought to always be deleterious, it has since been established that they have the potential to be beneficial under certain conditions, since neurotrophic factors have been detected in peripheral blood mononuclear cells (PBMCs) [17]. Indeed, production of the neurotrophic factors BDNF and NT-3 by immune cells has been shown to accompany better recovery from spinal cord injury [18], and the production of neurotrophic factors by immune cells has been hypothesized as a potential means of neuroprotection in MS [19].

Recently, a pilot clinical trial using testosterone to treat 10 male MS patients was completed [20]. In these patients, cognitive function improved and the brain atrophy rate was significantly slowed. Here, immunomodulatory effects of testosterone treatment as well as its ability to induce neurotrophic growth factor production in PBMCs were examined in these patients to explore a potential novel pathway underlying testosterone's beneficial effects.

## Methods

### Subjects

Ten men (mean age 46, range 29–61) who met Poser criteria for clinically definite relapsing-remitting (RR) MS, were not currently receiving disease modifying treatment and had a mean Expanded Disability Status Score (EDSS) of 2.0 (range 1.5–2.5) were studied. A six month pretreatment observation period (month -6 to 0) was followed by twelve months (months 1 to 12) of treatment with 10 g of gel containing 100 mg of testosterone (AndroGel^®^) applied to the upper arms once per day. Treatment increased circulating testosterone levels from the lower normal range to the higher range of normal, with average increases of 50% [20].

Every three months clinical examinations were done and blood was drawn to collect PBMCs. The protocol was approved by the UCLA Human Subjects Protection Committee and all patients provided written informed consent prior to enrollment in the study.

### Delayed type hypersensitivity (DTH) test

Delayed type hypersensitivity (DTH) recall responses to tetanus (Tetanus Toxoid; Wyeth Laboratories, Marietta, PA) were assessed at two time points: once in the pretreatment period at study month 0 and once in the middle of the treatment period at study month 6, as described [21].

### PBMC phenotyping and ex vivo stimulation

Cryopreserved PBMCs collected every three months throughout the pretreatment and treatment periods were thawed and analyzed simultaneously for every patient. PBMC subpopulations before (month 0) and at the end of treatment (month 12) were determined by flow cytometry. One aliquot of cells was stained for cell surface markers with a panel of conjugated Abs, including CD3 (FITC), CD64 (FITC), CD14 (PE), CD4 (PE), CD8 (PE), CD16+56 (PE), CD45 (PerCP) and CD19 (Per-CP) (BD Bioscience) to determine subpopulations.

For *ex vivo *protein production, PBMCs were cultured at 1 × 10^5 ^cells/well with either (1) anti-CD3 (1 μg/ml; Sigma-Aldrich, St. Louis, MO) and anti-CD28 (2.5 μg/ml; Sigma-Aldrich), or (2) PHA (5 μg/ml; Sigma-Aldrich) or (3) media alone, as described [22] Protein levels were assayed in supernatants at 48 h and 72 h using SearchLight multiplex assays for growth factors (brain-derived neurotrophic factor BDNF, platelet-derived growth factor PDGF-BB, nerve growth factor NGF, and ciliary neurotrophic factor CNTF) and cytokines (IL-2, IFNγ, TNFα, IL-17, IL-10, IL-12p40, TGFβ1).

### Primary neuronal cultures

Primary CNS cell cultures were prepared from P0 C57BL/6 pups. Forebrain tissue was removed, dissociated, and plated on poly-D-lysine coated 18 mm coverslips in 12-well culture plates. Dissociated brain tissue from littermates was pooled and plated at a density of 10,000 cell/ml in Neurobasal media (Gibco) containing 2% B27 supplement (Invitrogen), 1% Penicillin-Streptomycin, 0.5 mM L-glutamine, and 10 ng/ml NGF (Invitrogen). All experiments were done with the approval of the UCLA Committee for the Protection of Research Subjects. Cells were cultured for 10 days with media exchanged every three days. Then, cells were washed 2× in RPMI and exposed to 1 mM glutamate for 2 h in the presence or absence of growth factors to test the biological significance of the combination and dose of growth factors produced by immune cells. Brightfield microscopy images of TUNEL (Roche) stained culture slides were obtained at 40× to visualize neurons. Lactate Dehydrogenase (LDH) is a stable cytoplasmatic enzyme present in all cells, which is rapidly released into the cell culture upon damage of the plasma membrane and its quantification can be used as an objective measure of cell damage. LDH levels were measured with a commercially available Cytotoxicity Detection Kit (Roche) according to manufacturer's instructions at 490 nm on a Multiskan plate reader.

### Statistical analysis

For growth factor and cytokine levels, the two baseline values (month -3 and month 0) were averaged and all values were log-transformed to achieve a normal distribution. Changes in growth factor and cytokine levels were tested using repeated measures ANOVA including all stimulation conditions and incubation times. In case of a significant main effect for time in the ANOVA, exploratory pairwise comparisons were computed to determine significant increases over baseline. Changes in lymphocyte subpopulations, DTH recall test results and *in vitro *neurotoxicity results were compared using paired t tests. Pearson correlation coefficients were computed to examine the association between changes in production of cytokines and growth factors from baseline to 12-month of treatment and changes in DTH and clinical outcomes. A value of p < .05 was considered significant.

## Results

**Figure 1:**
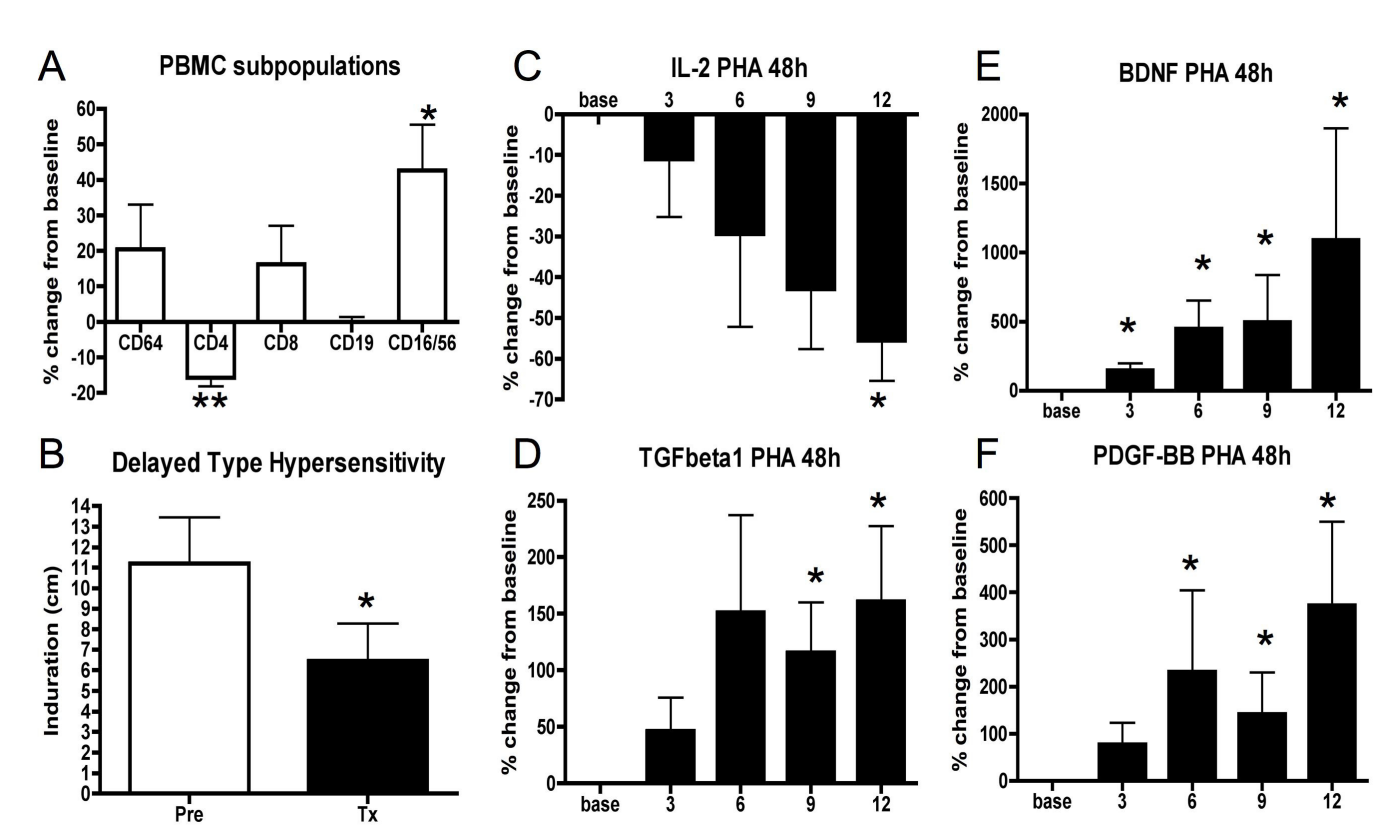
**Immunomodulation and growth factor induction by testosterone treatment in 10 male MS patients**. A, Testosterone treatment significantly decreased CD4+ T cell and increased CD16/56+ NK cell percentages. B, Treatment also significantly decreased delayed type hypersensitivity recall responses. C-F, In addition, treatment significantly decreased IL-2 and increased TGFβ1, BDNF and PDGF-BB levels produced by PHA stimulated peripheral blood mononuclear cells (PBMCs) during testosterone treatment (months 3–12) compared to baseline (base). Protein levels are expressed as the mean percent change compared with the mean from two pretreatment baseline time points. Mean month 12 concentrations were 670.5 ± 223.4 pg/ml for IL-2, 1552.0+273.3 pg/ml for TGFβ1, 246.1 ± 40.37 pg/ml for BDNF and 42.6 ± 15.6 pg/ml for PDGF-BB, respectively.

Testosterone treatment significantly decreased CD4 T cell populations (average decrease 17%, p = .03) while increasing NK cell populations (average increase 64%, p = .03, Figure 1A). Furthermore, DTH recall responses were significantly decreased during treatment compared to pre-treatment values (mean millimeters induration pre-treatment 11.2 ± 2.3; treatment 6.5 ± 1.8, p = .03, Figure 1B).

Regarding effects of testosterone treatment on cytokine levels, there was a significant decrease in IL-2 production during the treatment period as compared to pretreatment baseline (p < .001, Figure 1C). Average decreases in IL-2 were -34% for CD3/CD28 and -58% for PHA stimulation. In contrast, a significant increase was observed in TGFβ1 levels (p < .0001, Figure 1D) with average increases of 167% for CD3/CD28 and 179% over baseline for PHA stimulations. No significant changes were observed in other cytokines (IFNγ, TNFα, IL-10, IL-17, IL-12p40) tested.

Testosterone treatment significantly increased levels of growth factors in supernatants of *ex vivo *stimulated PBMCs. Specifically, BDNF levels in PBMC supernatants revealed average increases of over 9 fold at treatment month 12, as compared to pretreatment baseline, for CD3/CD28 stimulation, and almost 11 fold for PHA stimulation, (p < .0001, Figure 1E). Highly significant increases in PDGF-BB production were also observed during testosterone treatment, with PDGF-BB levels increasing approximately 3.5 fold during both CD3/CD28 and PHA stimulation conditions (p < .0001, Figure 1F). No significant changes were observed in other growth factors (NGF, CNTF) tested.

Correlation coefficients of cytokine and growth factor changes with functional and clinical outcomes were computed as an exploratory analysis. The increases in BDNF, PDGF-BB and TGFβ1 production were significantly correlated (TGFβ1 and BDNF r = .72; TGFβ1 and PDGF-BB, r = .89; BDNF and PDGF-BB, r = .91). There was a moderate (but not significant) correlation between TGFβ1 and IL-2 changes (r = .48). Interestingly, there was a positive association between the decrease in CD4+ cells and decreased responses in the DTH skin test (r = .59) that showed a statistical trend (p = .07). However, changes in IL-2 did not significantly correlate with either CD4+ percentage or DTH. As reported previously [20], testosterone treatment in our study increased cognitive function as measured by the Paced Auditory Serial Addition Task (PASAT). Percent increases in BDNF (r = .74, p = .01) and PDGF-BB (r = .73, p = .02) from baseline to month 12 of treatment were significantly associated with improvements in PASAT testing during the same time. However, the associations with the PASAT were driven by the two strongest responders in growth factors who also had the strongest improvement in cognitive function and should thus be interpreted with caution.

**Figure 2:**
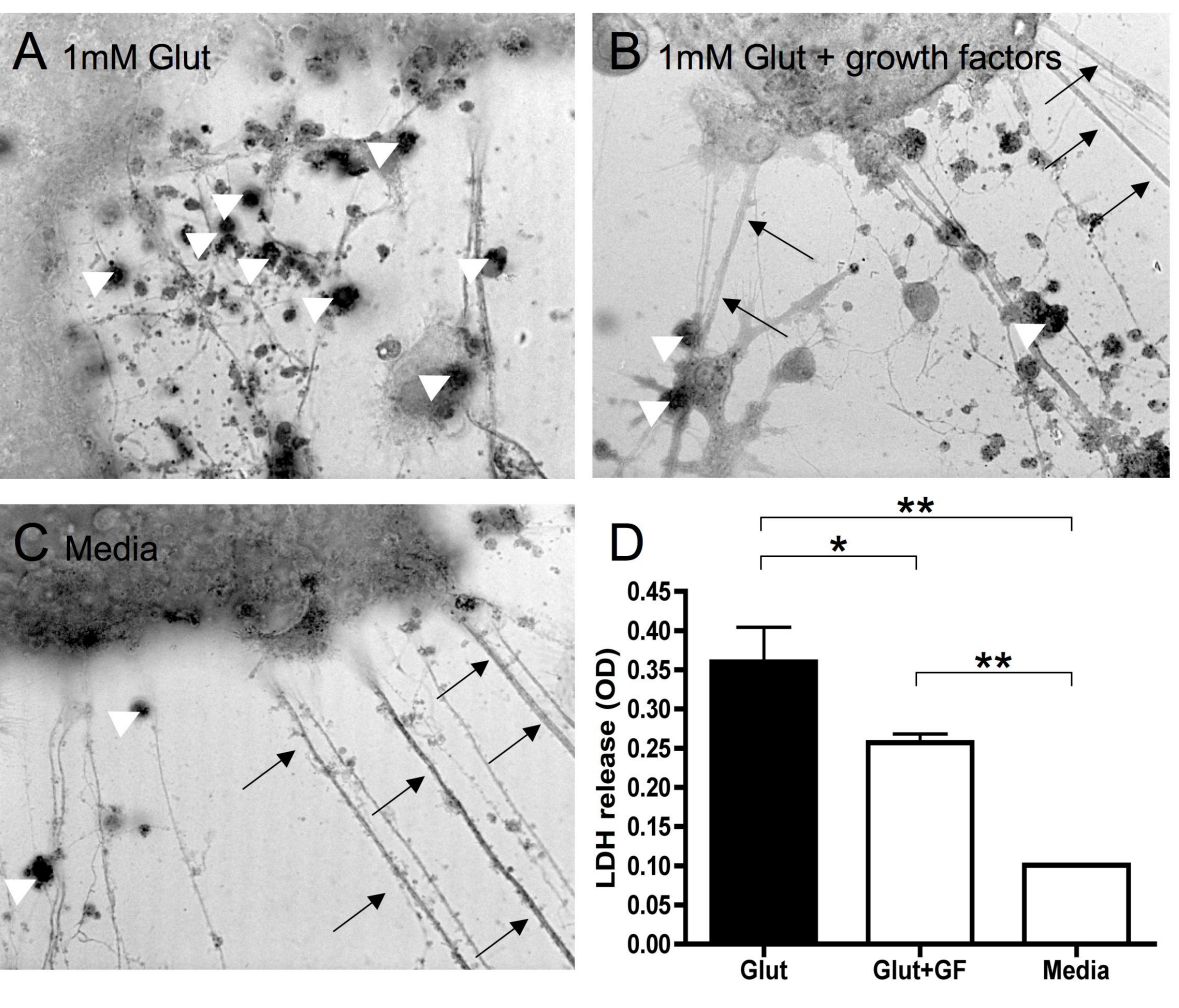
**Growth factors reduce neurotoxicity in vitro**. Neuroprotection against glutamate toxicity (1 mM for 2 h) by growth factors at concentrations similar to those at treatment month 12 in the clinical trial (2000 pg/ml TGFβ1, 500 pg/ml BDNF, 100 pg/ml PDGF-BB). A-C, Brightfield microscopy images indicate that addition of growth factors during glutamate exposure induced partial protection of axonal integrity in those cultures (black arrows) and decreased the number of TUNEL positive cells (white triangles), representative images shown from 8 experiments. D, a protective effect was confirmed quantitatively by a significant reduction in LDH release. LDH data are shown from 8 independent experiments with wells run in duplicate for each condition.

Next, we aimed to determine whether levels of BDNF, PDGF-BB, and TGFβ1, which were induced by *in vivo *testosterone treatment, were functionally significant. Glutamate excitotoxicity is a widely used model for neurotoxicity and is thought to contribute to neurodegeneration observed in MS [23]. Thus, to test the biological significance of growth factor levels produced by immune cells during treatment, neuronal cultures were exposed to 2 h of 1 mM glutamate in the presence or absence of recombinant growth factors similar to the levels seen in the best treatment responder at month 12 (2000 pg/ml TGFβ1, 500 pg/ml BDNF, 100 pg/ml PDGF-BB). Brightfield microscopy images indicated that addition of growth factors induced partial protection of axonal integrity in those cultures (black arrows) and decreased the number of TUNEL positive cells (white triangles, Figure 2A–C). LDH quantification showed that the addition of growth factors during glutamate exposure significantly reduced LDH release from neuronal cultures, in line with a neuroprotective effect (p = .04, Figure 2D).

## Discussion

Our data suggest that testosterone treatment in MS is associated with effects on markers that could play a role in the inflammatory as well as the neurodegenerative component of the disease. A decrease in DTH responses, decreased IL-2 and increased TGFβ1 production suggest an anti-inflammatory effect. This was accompanied by a decrease in CD4+ cells and an increase in NK cells. Based on our current understanding of MS pathogenesis, CD4+ autoreactive T cells and their differentiation into a Th1 phenotype are crucial events in disease development, and these cells are probably also important players in the long-term evolution of the disease [24], while NK cells have been suggested to play a regulatory role by killing myelin-specific T cells and immature dendritic cells, promoting regulatory T cells and enhancing Th2-like responses [25].

Both IL-2 and TGFβ1 have been proposed as treatment targets in MS. Blocking the IL-2α receptor chain with the monoclonal antibody daclizumab has been shown to decrease active lesions on MRI [26]. Interestingly, daclizumab treatment is also accompanied by a decrease in CD4+ T cells, as well as an increase in a subpopulation of NK cells numbers [27], consistent with our results on alterations in lymphocyte subpopulations during testosterone treatment.

The role of TGFβ1 in the immune system is complex as it can affect multiple cell lineages, either promoting or opposing their differentiation, survival, and proliferation. However, TGFβ1 appears to be a critical modulator of T cell-mediated self-reactivity [28] and has been successfully used to treat EAE [29]. Unfortunately, a clinical trial of TGFβ2 administration in secondary-progressive MS had to be stopped due to nephrotoxicity of the recombinant cytokine [30].

In addition to these immunomodulatory effects, we saw significant increases in growth factor production by PBMCs. Neuroprotective effects of testosterone treatment have been described using both *in vitro *cultures and *in vivo *models. Since testosterone is lipophilic, *in vivo *effects of systemic testosterone treatment are thought to be mediated by testosterone crossing the blood brain barrier. In the CNS, testosterone may bind androgen receptors or may be converted by aromatase to estrogen, with estrogen known to have neuroprotective properties [31]. Here, we describe a novel potential neuroprotective pathway of testosterone treatment, namely through the induction of BDNF, PDGF-BB, and TGFβ1 by peripheral blood immune cells.

This is relevant to MS since neurotrophic factors such as BDNF have been detected in infiltrating immune cells in CNS lesions in both experimental autoimmune encephalomyelitis (EAE) [32] and MS [33] and it has been suggested that the immune cell-mediated import of BDNF and other neurotrophic factors into the central nervous system has the functional relevance of curbing the detrimental effects of inflammation on the surrounding tissue [19]. Supporting the clinical relevance of this phenomenon, an increase in BDNF production by PBMCs during relapse was previously only observed in patients who achieved full recovery, while patients with incomplete recovery demonstrated no alterations in BDNF production [34]. In addition, a recent study has shown correlations of BDNF production by peripheral immune cells with MRI-based measures of disease severity. Lower BDNF production was associated with lower Magnetic Transfer Ratio (MTR) in normal appearing white matter, suggesting more white matter damage in patients with lower BDNF production [35].

The neuroprotective effects *in vivo *and *in vitro *of BDNF are well known [36]. PDGF-BB has also been shown to be neuroprotective *in vitro *[37] and *in vivo *[38]. It is interesting to note that TGFβ1, in addition to its anti-inflammatory effects, also protected neurons from glutamate toxicity *in vitro *and *in vivo *[39]. Supporting the protective potential of growth factors produced by PBMCs in our study, we found that a combination of recombinant growth factors at similar concentrations as seen at month 12 of treatment could reduce glutamate-induced neurotoxicity *in vitro*. In this regard, it is intriguing that when the subjects in our study were assessed for MRI and clinical outcomes, a slowing in brain atrophy and an increase in cognitive function was observed during testosterone treatment as compared to baseline [20].

## Conclusion

Previously, clinical trials using neurotrophic factors have been disappointing in neurodegenerative diseases despite promising results from pre-clinical studies [40]. The negative results of these clinical trials have highlighted that the manner and site of administration are of critical importance. Similarly, anti-inflammatory strategies in MS using monoclonal antibodies or recombinant cytokines have been associated with either partial efficacy or significant toxicities.

In contrast, treatment with physiological levels of testosterone is safe and appears to orchestrate simultaneous alterations in multiple growth factors and cytokines with the delivery system being the subjects' own peripheral blood immune cells. This immunomodulatory and potential neuroprotective pathway warrants further study in MS and other neurodegenerative diseases, which contain an inflammatory component, namely Alzheimer's Disease, Parkinson's disease and spinal cord injury [41].

## List of abbreviations

BDNF: Brain-derived neurotrophic factor; CNTF: Ciliary neurotrophic factor; DTH: Delayed type hypersensitivity; EAE: Experimental autoimmune encephalomyelitis; EDSS: Expanded disability status scale; IL: Interleukin; LDH: Lactate Dehydrogenase; MRI: Magnetic resonance imaging; MS: Multiple sclerosis; NGF: Nerve growth factor; NK cell: Natural killer cell; NT-3: Neurotrophin 3; PASAT: Paced Auditory Serial Addition Task; PBMCs: Peripheral blood mononuclear cells; PDGF: Platelet-derived growth factor; PHA: Phytohemagglutinin; RRMS: Relapsing-remitting MS; TGF: Transforming growth factor; TNF: Tumor necrosis factor.

## Competing interests

The authors declare that they have no competing interests.

## Authors' contributions

SMG contributed to study design and concept, acquired the immunological data, performed the neurotoxicity assay, and was responsible for analysis, interpretation and preparation of the manuscript. SC contributed to the neurotoxicity assay and critically revised the manuscript. BSG was responsible for patient assessment and acquisition of the clinical data and critically revised the manuscript. RRV obtained funding, was responsible for study design and concept and contributed to interpretation and preparation of the manuscript. All authors have read and approved the final manuscript.
